# Distribution patterns of the two genetic groups of *Corbicula fluminea* in a lotic–lentic system

**DOI:** 10.1002/ece3.11339

**Published:** 2024-05-20

**Authors:** Meixiang Jia, Fei Cheng, Jin Li, Bjorn Victor Schmidt, Yacheng Li, Songguang Xie

**Affiliations:** ^1^ The Key Laboratory of Aquatic Biodiversity and Conservation of Chinese Academy of Sciences Institute of Hydrobiology, Chinese Academy of Sciences Wuhan China; ^2^ University of Chinese Academy of Sciences Beijing China; ^3^ School of Marine Biology and Fisheries Hainan University Haikou China; ^4^ Key Laboratory of Southwest China Wildlife Resources Conservation, Ministry of Education China West Normal University Nanchong China; ^5^ Department of Biological and Environmental Sciences Texas A&M University‐Commerce Commerce Texas USA; ^6^ Hongze Lake Fisheries Administration Committee Office of Jiangsu Province Huaian China

**Keywords:** genetic divergence, habitat preference, morphotype, shell sculpture, the Hongze Basin

## Abstract

Differences in local habitat conditions are often implicated as drivers for morphological and genetic divergence in natural populations. However, there are still relatively few studies regarding how divergent habitats influence patterns for morphotypes and genetic lineages in aquatic invertebrates. In this study, we explored the morphological patterns, genetic divergence, and distributions of a bivalve, *Corbicula fluminea*, in a lotic–lentic system. Sampling locations included lotic, ecotone, and lentic habitats. First, we found two lineages (Lineages A and B) with significant genetic divergence that primarily corresponded to two morphotypes (Morphs D and C) of *C. fluminea*. Lineage A consisted of 88.68% Morph D (shell sculpture: 8–14 ridges/cm_sh_) and 11.32% Morph C (shell sculpture: 15 ridges/cm_sh_) individuals and had genetic similarity to invasive populations. Lineage B consisted of only Morph C (shell sculpture: 15–23 ridges/cm_sh_). Second, we revealed clear effects of habitat on the spatial distribution patterns for the two lineages of *C. fluminea*. Lineage A was dominant in lotic habitats, with a significantly higher density than that of Lineage B in these locations. Lineage B was dominant in lentic habitats. However, both lineages had their highest densities in the ecotone habitat, without clear dominance and no significant difference in density between groups. Individuals of Lineages A and B are different in shell morphology, which may be related to a benefit trade‐off between shell shapes that allow for rapid burrowing and holding position in different flow conditions. The distribution patterns indicate that Lineages A and B may not prefer uniquely lotic and lentic habitats, but each lineage is more tolerant to one habitat type, respectively. Generally, our study established a correlation among morphotypes, lineages, and different habitats for *C. fluminea* along a lotic–lentic gradient system, which has important implementations for fisheries management units and for understanding the role of habitat preference for this species in monitoring for pioneer dispersal in invasive species management.

## Graphical abstract

Habitat environments driving population divergence are a core idea of the ecological speciation hypothesis. However, little is known regarding this hypothesis in invertebrates. This study validated the morphologically and genetically diverging groups with different habitat preferences for *Corbicula fluminea* in a lotic–lentic system, which suggests a highly valuable invertebrate model for the ecological speciation hypothesis. Our results also have important implications for the management of fishery resources and invasive populations.

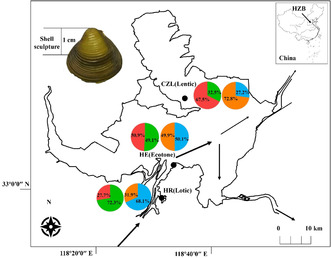

## 1 INTRODUCTION

Morphological and genetic divergence is ubiquitous in natural populations, often forming from similar or the same ecological factors (Nosil, 2012; Rundle & Nosil, 2005; Schluter, 2001). One important driver that can influence these patterns is differing local habitat conditions (Rundle & Nosil, 2005; Schluter & Conte, 2009). Among aquatic animals, there is ample empirical and theoretical evidence obtained from fish populations in differing lotic and lentic systems that support these correlations (Brawand et al., 2014; Ostberg et al., 2009; Theis et al., 2014). Compared to lentic habitats in lakes, lotic habitats in rivers generally have a faster water velocity, higher spatial heterogeneity and oxygen concentration, and larger size of substrate particles (Thorp et al., 2001). Through adaptation to different habitat conditions, morphological divergence between lotic and lentic populations is common and generally consistent across all fish taxa (Theis et al., 2014). For example, populations of fish (*Siphateles bicolor snyderi*) coping with higher water velocity in rivers commonly have more streamlined bodies with narrow, elongated, and high surface ratios of the caudal fin to help in maintaining a steady position in the presence of stronger flow conditions (Baker et al., 2021). Other morphological divergence, such as increasing and decreasing gill size under hypoxic and normoxic conditions, have also been reported between lotic and lentic populations of African cichlid fish (*Pseudocrenilabrus multicolor victoriae*) (Crispo & Chapman, 2010). Genetic divergence between lotic and lentic fish populations is also common when populations have geographic isolation (Rennison et al., 2019; Reusch et al., 2001). Moreover, morphological and genetic divergence can occur among sympatric populations occupying different local habitats, such as benthic and limnetic populations of stickleback fish (*Gasterosteus aculeatus* L., 1758 complex) (Härer et al., 2021; McGee et al., 2013).

Correlations between habitat divergence and morphological and genetic patterns are well established in fish, which have been helpful to understand potential drivers of fish diversity patterns and to design management measures. However, the influence of lotic and lentic habitats on morphotypes and genetic divergence has not been well established for *Corbicula fluminea*, one of the most invasive bivalves globally, which is a drawback in understanding how habitat‐related population and genetic patterns can be used for formulating prevention and control management of this invasive species (Ilarri et al., 2019; Karatayev et al., 2005; Li et al., 2022; Zeng et al., 2023). *Corbicula fluminea* has successfully invaded aquatic systems in America and Europe that contain both lotic and lentic local habitat conditions (Bespalaya et al., 2018; Crespo et al., 2017; Haponski & Foighil, 2019), with a total of seven morphotypes being reported from different invasion locations (Appendix S1), specifically four morphotypes (Forms A–D) in America and three (Forms R, Rlc, and S) in Europe (Haponski & Foighil, 2019; Marescaux et al., 2010). The highly invasive potential of *C. fluminea* may be related to its capacity in coping and succeeding in different habitats through variable morphologies represented in these morphotypes (Hünicken et al., 2022). The bivalve can inhabit heterogeneous conditions from freshwater to seawater (Bertrand et al., 2017) and is successful in lentic and lotic habitats (Patrick et al., 2017). Different ecological impacts have also been revealed from the production of diverse morphotypes in these heterogeneous habitats (Bespalaya et al., 2018; Penarrubia et al., 2017; Pigneur et al., 2011). Morphological divergence of *C. fluminea* populations have been found mainly in its shell shape, which may be related to different habitat factors, such as water velocity, substrate sediment, and nutrient loadings (Stiven & Arnold, 1995). Morphotypes in lotic habitats generally have a more asperous shell that protects against displacement risk from higher water velocities, and those types in lentic habitats typically have a more smooth shell to gain advantages in burrowing, suggesting that water velocity (lotic–lentic gradient) is a critical factor in the shell variations of the bivalve (Thorson et al., 2017; Zhou et al., 2011). Furthermore, genetic studies of invasive populations of *C. fluminea* have determined a total of five genetic lineage groups with strong patterns of genetic divergence between lineages (Haponski & Foighil, 2019). Obviously, because there are more invasive morphotypes than lineages, some distinct invasive morphotypes share the same or similar genetic backgrounds (Gomes et al., 2016; Kijviriya et al., 1991; Sousa et al., 2007). Knowledge of morphological and genetic compositions and their correlation with specific habitat conditions will help to evaluate invasion risks/effects for specific systems and to design priority for prevention, detection monitoring, and control managements of *C. fluminea*.

Additionally, *C. fluminea* is an important fishery resource in East Asia, which is one of its native distribution areas (Bi et al., 2014; Li et al., 2022). Diverse morphological and genetic groups have also been found from these native populations (Wang et al., 2014; Wu et al., 2019). However, fishery management in native populations of the bivalve usually does not integrate these different groups into management plans, which may be due to lack of a distinct correlation between morphotypes and genetic lineages. For example, the bivalve is an important composition of fisheries in the Hongze Lake, China, where the annual harvest is approximately 2 × 10^4^ t, supporting an artisanal fishery around the lake (Hongze Lake Fisheries Administration Committee Office of Jiangsu Province, unpublished data). Our previous study identified two morphotypes (Morphs C and D, Appendix S1) with significant genetic differentiation based on the shell sculpture of *C. fluminea* in the lake (Li et al., 2022). Furthermore, results suggested that Morph C may have higher density in low‐flow environments, whereas Morph D may be competitive in high‐flow environments (Li et al., 2022). However, fishery management of the bivalve in the Hongze Lake has long been based on a single management unit, which might result in failure of resource management (Bi et al., 2014).

Overall, a correlation among morphotypes, genetic lineages, and lentic‐to‐lotic habitats has not been well established for *C. fluminea*, which may be an impediment to advise measures for the prevention and control of invasive populations and for fishery management of native populations. In this study, we wanted to test specifically if identified morphotypes of *C. fluminea* in the Hongze Lake are associated with unique genetic lineages and have different habitat preferences along lotic‐to‐lentic gradients. To test this hypothesis, we collected *C. fluminea* specimens from lotic, ecotone, and lentic habitats within the Hongze Basin, identified their morphological and genetic composition, and tested the ratio of divergent morphotypes and lineages along the lotic–lentic gradient in the system.

## 2 MATERIALS AND METHODS

### 2.1 Study areas

**FIGURE 1:**
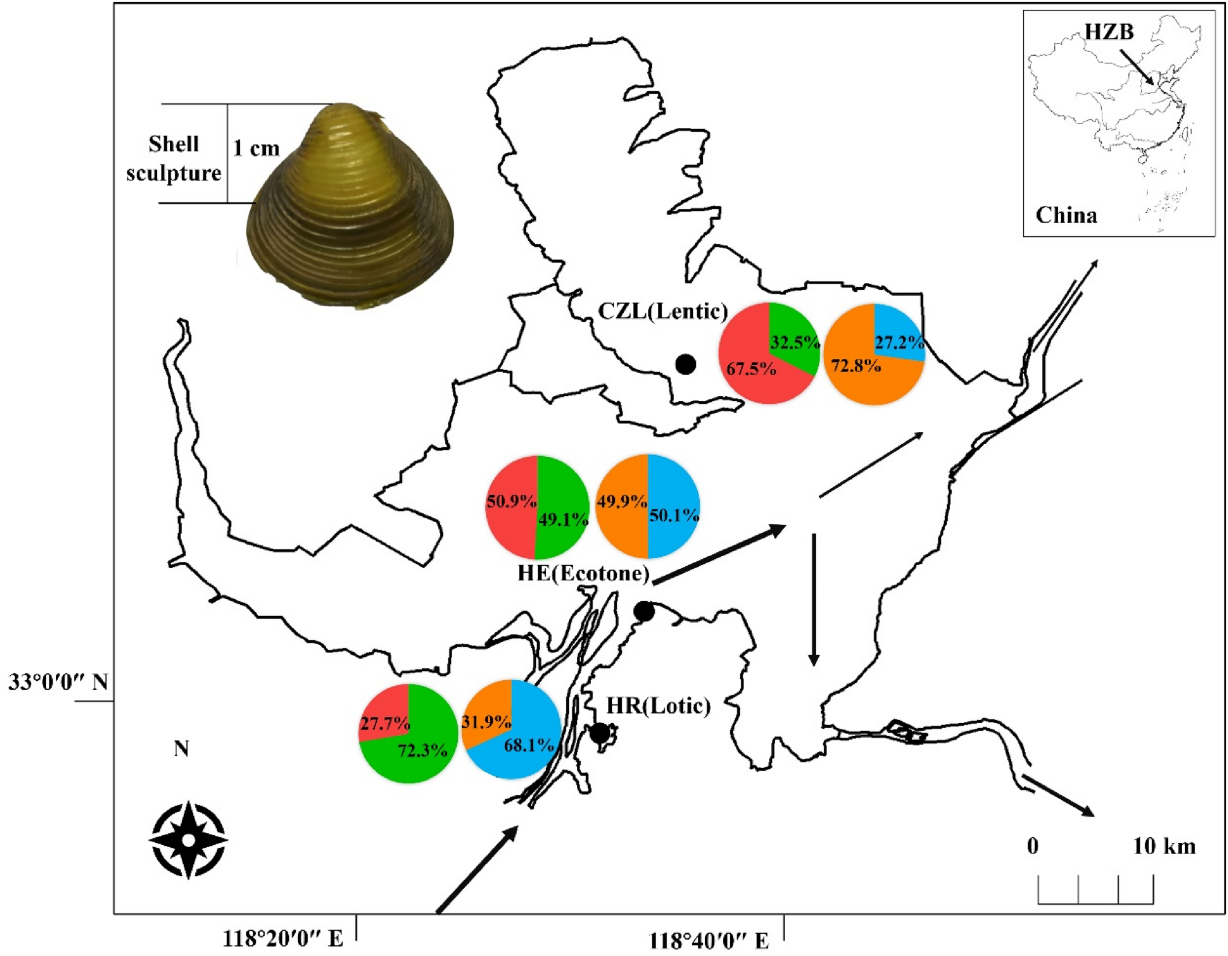
Map of the Hongze Basin (HZB), which is a typical lotic and lentic gradient system. Sampling sites for *Corbicula fluminea* included the Huaihe River (HR), the Huaihe Estuary (HE), and Chengzi Lake (CZL). The arrow indicates the direction of major flow in the lake and the thickness of the line indicates the flow magnitude. The shell sculpture (ridge count/cm_sh_) was measured as the number of ridges within 1 cm of shell height, which is showed using Morph D sculpture. Density ratios between Lineages A (green) and B (red), and between Morphs C (orange) and D (blue) of *C. fluminea* in the HR, the HE, and the CZL are shown in different colors.

The Hongze Lake (33°06′–33°40′ N, 118°10′–118°52′ E) is the fifth‐largest freshwater lake in China with an area of 1576.9 km^2^ (Deng et al., 2018). The Huaihe River flows into the southwestern part of the lake and contributes approximately 70% of its annual water input (Lei et al., 2020). Water flows out from the southern and eastern banks of the lake (Figure 1). The hydrographic conditions in the lake create highly heterogeneous environments with regard to flow (Ye et al., 2011). For example, both the inflow and outflow points are located away from the northern part of the lake, which therefore has a relatively lentic habitat. Combined with the largest tributary, the lotic and lentic habitat gradient in the Hongze Basin provides a valuable model for studying the correlation among morphotypes, genetic lineages, and habitat preferences of *C. fluminea* populations. Three sampling habitats were selected in this study: the main stem of the Huaihe River (HR, lotic habitat), the Huaihe Estuary (HE, ecotone), and the Chengzi Lake (CZL, lentic habitat) (Figure 1).

### 2.2 Sampling and environmental data collection

*Corbicula fluminea* were collected from each habitat in July, 2023. Sampling was conducted using a dredge, which is also used by local fishermen for commercial catching. The dredge used had a 6 mm mesh, was 70 cm wide, 50 cm long, and 10 cm in height. When sampling, the dredge dug into the sediment with approximately 10 cm depth and was pulled for a distance of 150 m. Samples were collected, washed, and then transported to the laboratory (Li et al., 2022).

Data on nine environmental variables were collected from each sampling habitat using conventional methods. Habitat parameters, including water temperature, water depth, water velocity, transparency, dissolved oxygen, conductivity, pH, turbidity, and salinity, were all measured immediately before sampling (Appendix S2). The HR had clearly different dissolved oxygen concentrations and transparency compared to the other habitats; the CZL had different values for pH, turbidity, water velocity, and salinity, and the variables of the HE indicated the environmental conditions of a typical ecotone between the lake and river (Appendix S2). These variables showed that the sampling sites had different environmental characteristics, with typical lotic habitat conditions in the HR and relatively lentic habitat conditions in the CZL.

### 2.3 Morphological and genetic analysis

We measured the shell sculpture of all collected specimens from the three sampling habitats. The inner organs of each specimen were removed. The shells were washed and dried under natural conditions before the procedure. The shell sculpture (ridge count/cm_sh_) of each individual was measured as the number of ridges within 1 cm of shell height (Bodis et al., 2011; Li et al., 2022; Pfenninger et al., 2002). The morphometric region for the shell sculpture is shown in Figure 1. Individuals were assigned to two morphotypes based on their shell sculpture, namely Morph D for individuals with <15 ridges/cm_sh_ and Morph C for those individuals with ≥15 ridges/cm_sh_ (Li et al., 2022).

Thirty specimens from each habitat were randomly selected for genetic analyses. A total of 89 specimens were successfully amplified, which included 42 Morph C and 47 Morph D individuals (Appendix S3). Soft tissues of each specimen were carefully collected, immediately preserved in 95% ethanol, and stored at 4°C. Total genomic DNA was extracted using an Animal Genomic DNA Kit (TsingKe, Wuhan, China). The mitochondrial DNA (mtDNA) *CO I* gene fragment was amplified using the universal DNA primers LCO1490 and HCO2198 through polymerase chain reaction (PCR) (Folmer et al., 1994). The PCR was performed, as described by Park et al. (2002). The amplicons were purified and sequenced by a commercial sequencing company (TsingKe, Wuhan, China).

Sequences were visualized and aligned using Chromas 2.5 (Yuan et al., 2015). Genetic lineages of *C. fluminea* were determined, according to the haplotype evolution network diagram constructed using the median‐joining method of Network10 (Kong et al., 2016). Polymorphic indices (number of haplotypes, polymorphic sites, haplotype diversity, nucleotide diversity, and average number of nucleotide differences) of each identified lineage and morphotype were calculated using DnaSP6 (Librado & Rozas, 2009). The neighbor‐joining (NJ) tree and Kimura‐2 parameter genetic distance (K2P distance) between/among the identified groups were analyzed using MEGA7 (Molecular Evolutionary Genetics Analysis Version 7) (Tamura et al., 2011). The genetic differentiation coefficient (F_ST_) and the analysis of molecular variance (AMOVA) between/among the identified lineages and morphotypes were calculated using Arlequin version 3.0 (Excoffier et al., 2005); statistical significance was set at *p* < .05.

### 2.4 Distribution analysis

For distribution analysis, density (ind./m^2^) of the identified lineages and morphotypes was delineated for each sampling habitat. The density was calculated using the total catch obtained by pulling the dredge a distance of 150 m. In each sample, the density was calculated as the number of individuals collected divided by the area (0.7 m in width × 150 m in distance). We then calculated the density ratio of the different lineages and morphotypes in each habitat using this total. As the statistical conditions of normality for distributions and homogeneity of variances were not met, ratio differences between/among the lineages and morphotypes across habitats were, respectively, compared using a non‐parametric Kruskal–Wallis test in SPSS Statistics 21 (Wang et al., 2023). Statistical significance was set at *p* < .05.

## 3 RESULTS

### 3.1 Morphological and genetic analyses

**FIGURE 2:**
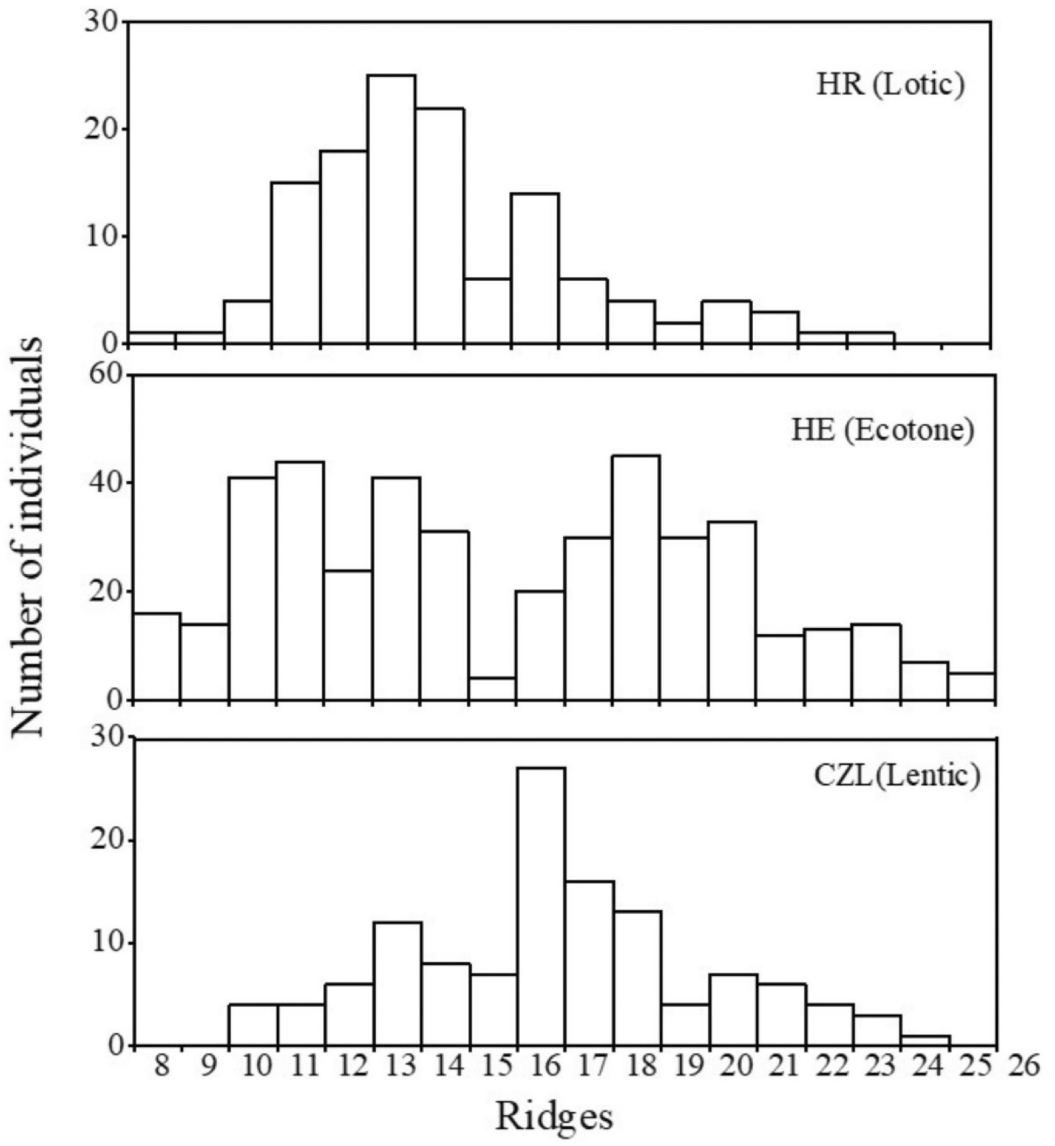
Frequency distribution histogram of the shell sculpture (ridge count/cm_sh_, the count of ridges within 1 cm in the direction of shell height) of *Corbicula fluminea* in the Huaihe River (HR), the Huaihe Estuary (HE), and the Chengzi Lake (CZL) of the Hongze Basin.

A total of 675 individuals were collected with the shell sculpture ranging from 8 to 25 ridges/cm_sh_ and assigned to two morphotypes (Morph C: 344 individuals, Morph D: 331 individuals). Means ± standard deviation (± SD) of the shell sculpture were 15.28 ± 4.04 ridges/cm_sh_ for all individuals, 18.64 ± 2.46 ridges/cm_sh_ for Morph C individuals, and 14.05 ± 2.85 ridges/cm_sh_ for Morph D individuals. A total of 127, 424, and 124 individuals were collected from HR, HE, and CZL, respectively. In HR, the population was dominated by Morph D (68.13%), in HE, population densities were almost equal between Morphs D (49.91%) and C (50.09%), and in CZL, populations were dominated by Morph C (72.76%) (Figure 1). The frequency distribution of the shell sculpture showed a unimodal pattern in HR and CZL and a bimodal pattern in HE. Means (± SD) of the shell sculpture were 14.05 ± 2.85 ridges/cm_sh_ in HR, 15.30 ± 4.42 ridges/cm_sh_ in HE, and 16.45 ± 3.31 ridges/cm_sh_ in CZL (Figure 2).

**FIGURE 3:**
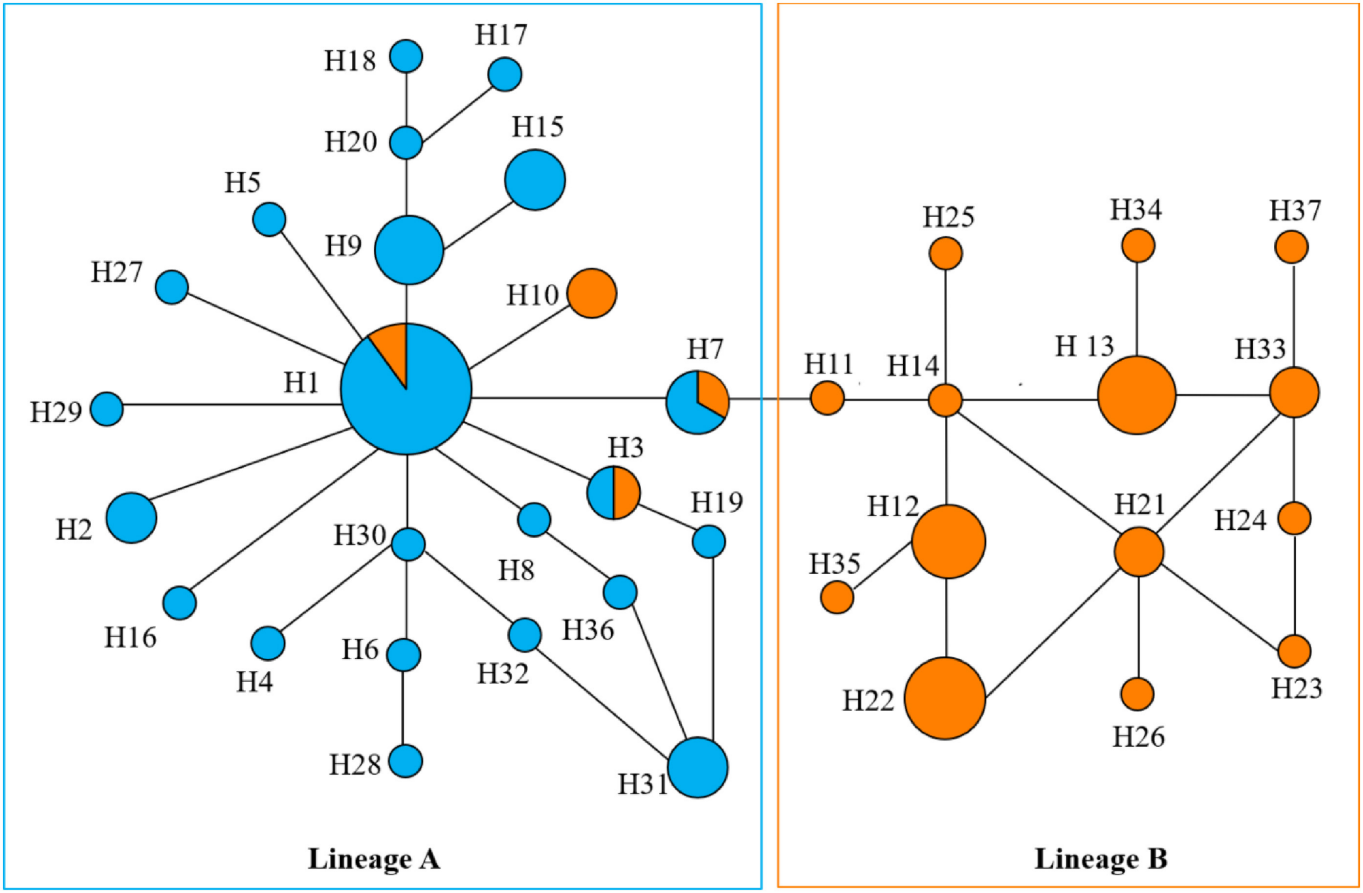
Haplotype evolution network diagram of *Corbicula fluminea* in the Hongze Basin using the median‐joining method by Network10. A total of 37 haplotypes are shown and named using H followed by a number; Lineages A and B were inferred according to the network; the circle represents a haplotype in the diagram; the size of the circle represents the individual number of individuals sharing the same haplotype, and color of the circle represents the morphotype (Morph C: orange, Morph D: blue).

A total of 37 haplotypes were detected from *CO I* gene fragment analysis. The haplotype evolutionary networks showed two separate evolutionary groups: designated as Lineages A and B (Figure 3). Lineage A had 23 haplotypes and consisted of 53 individuals including 47 Morph D and six Morph C. The shell sculpture of these six individuals was 15 ridges/cm_sh_. Thus, the shell sculpture of Lineage A consisted of 8–14 ridges/cm_sh_ (Morph D) and 15 ridges/cm_sh_ (Morph C) (Table 1). Lineage B had 14 haplotypes and consisted entirely of 36 Morph C individuals. The shell sculpture of Lineage B ranged from 15 to 23 ridges/cm_sh_ (Table 1).

**TABLE 1:** Relationship between the shell sculptures and genetic lineages of *Corbicula fluminea* of the Huaihe River (HR), the Huaihe Estuary (HE), and the Chengzi Lake (CZL) in the Hongze Basin.

Ridges	HR		HE		CZL		Total	
	Number	Lineage	Number	Lineage	Number	Lineage	Number	Lineage
8	1	A	1	A			2	A
9	1	A	1	A			2	A
10	1	A	2	A	1	A	4	A
11	1	A	4	A	1	A	6	A
12	3	A	4	A	2	A	9	A
13	4	A	5	A	5	A	14	A
14	4	A	1	A	5	A	10	A
15	2	B	2	B	6	A	10	A: 6, B: 4
16	3	B	5	B	1	B	9	B
17	3	B	2	B	1	B	6	B
18	2	B	1	B	2	B	5	B
19	2	B	1	B	1	B	4	B
20	1	B	1	B	2	B	4	B
21	1	B			1	B	2	B
22					1	B	1	B
23	1	B					1	B

*Note*: A: lineage A, B: lineage B.

**FIGURE 4:**
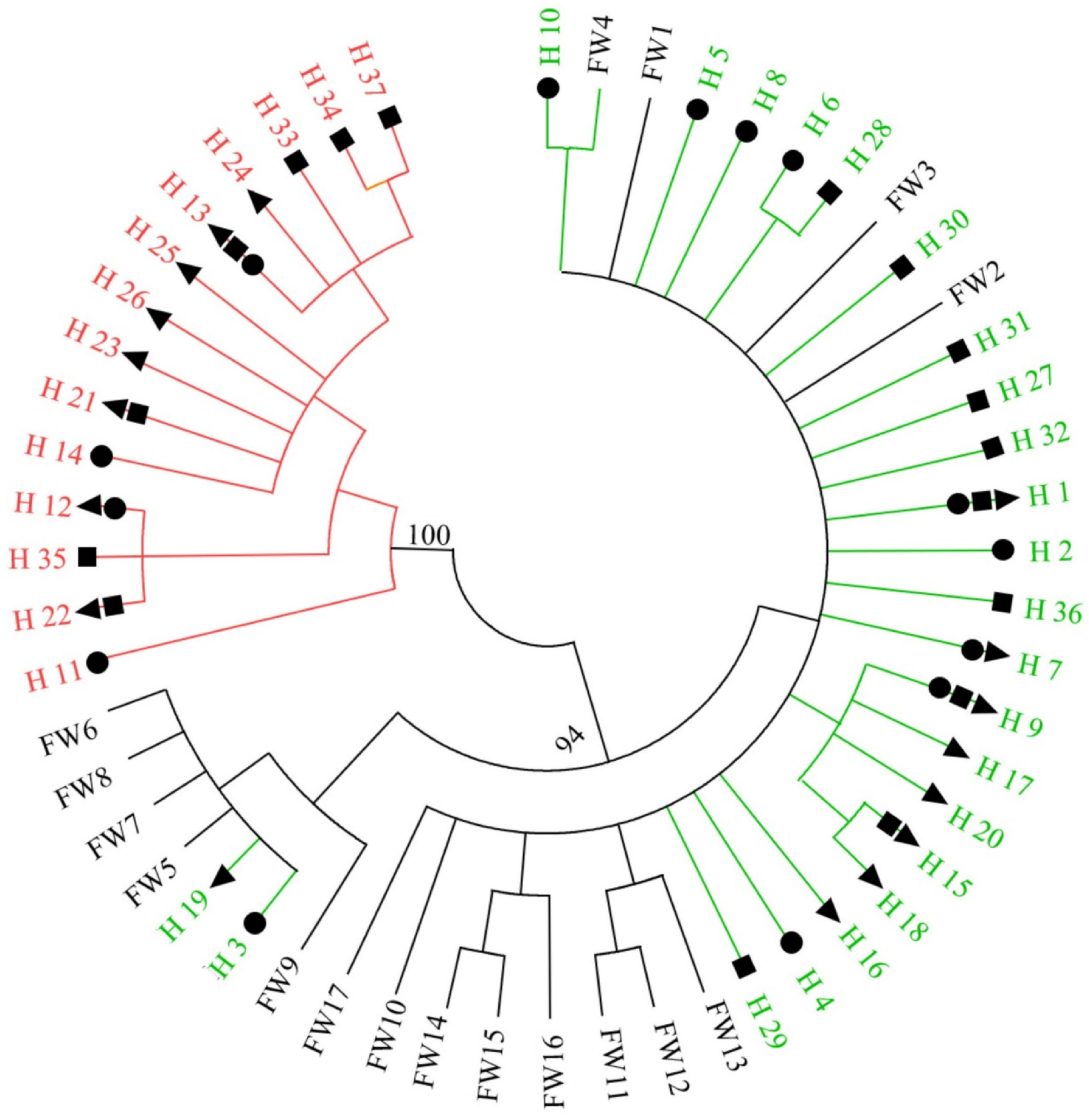
Neighbor‐joining tree inferred from haplotypes of *CO I* sequence fragments among Lineage A, Lineage B, and invasive populations. Different colors indicate different origins of haplotypes, green for haplotypes of Lineage A, red for haplotypes of Lineage B, and black for haplotypes reported from invasive populations (FW1–FW17); numbers (100 and 94) on the line represent the confidence level of the branches. Triangle, square, and circle represent haplotypes from the Huaihe River (Lotic), the Huaihe Estuary (Ecotone), and the Chengzi Lake (Lentic). Haplotypes without symbols represent the haplotypes of invasive populations.

Two distinct genetic clusters were revealed by neighbor‐joining (NJ) analysis of 54 haplotypes, including 17 haplotypes reported in invasive populations (Figure 4). Haplotypes from Lineage B clustered in a separate branch in the analysis, whereas the other branch included all haplotypes from Lineage A and the invasive populations (Figure 4).

Lineage A had higher genetic diversity values than Lineage B (Appendix S3). The K2P distance was 9.23% between Lineages A and B, and 1.00% within the lineages. Pairwise F_ST_ was 0.95 between Lineages A and B, indicating significant genetic divergence (*p* < .05). This divergence was confirmed by AMOVA, which showed 93.54% of genetic variation as being attributed to differences between the lineages (Table 2).

**TABLE 2:** Analysis of molecular variance (AMOVA) between Lineages A and B and between Morphs C and D of *Corbicula fluminea* in the Hongze Basin.

Group	Source of variation	*df*	Sum of squares	Variation components	Percentage of variation
Lineage A and Lineage B	Among populations	2	1202.09	24.36 Va	93.54
	Within populations	86	144.57	1.68 Vb	6.46
	Total	88	1346.66	26.04	
Morph C and Morph D	Among populations	1	919.80	20.62 Va	80.78
	Within populations	87	426.87	4.91Vb	19.22
	Total	88	1346.66	25.54	

### 3.2 Distribution of *C. fluminea* in different habitats

The density of *C. fluminea* was the highest in HE and the lowest in HR (Table 3). The density of Lineage A was higher than that of Lineage B in HR; the opposite density pattern for Lineages A and B was found in CZL; and the densities of Lineages A and B were similar in HE (Table 3). Morph D had the same distribution pattern as that of Lineage A, and the pattern of Morph C was the same as that of Lineage B (Table 3).

**TABLE 3:** Density (mean ± SD, × 10^−2^ ind./m^2^) of Lineages A and B, and Morphs C and D of *Corbicula fluminea* in the Huaihe River (HR), the Huaihe Estuary (HE), and the Chengzi Lake (CZL) of the Hongze Basin.

	Lineage A	Lineage B	Morph C	Morph D
HR	5.84 ± 2.06	2.22 ± 0.93	2.60 ± 0.98	5.46 ± 1.71
HE	30.38 ± 5.91	28.89 ± 7.89	29.49 ± 7.40	29.73 ± 5.77
CZL	5.58 ± 1.69	11.29 ± 1.39	12.24 ± 1.86	4.63 ± 1.02

**FIGURE 5:**
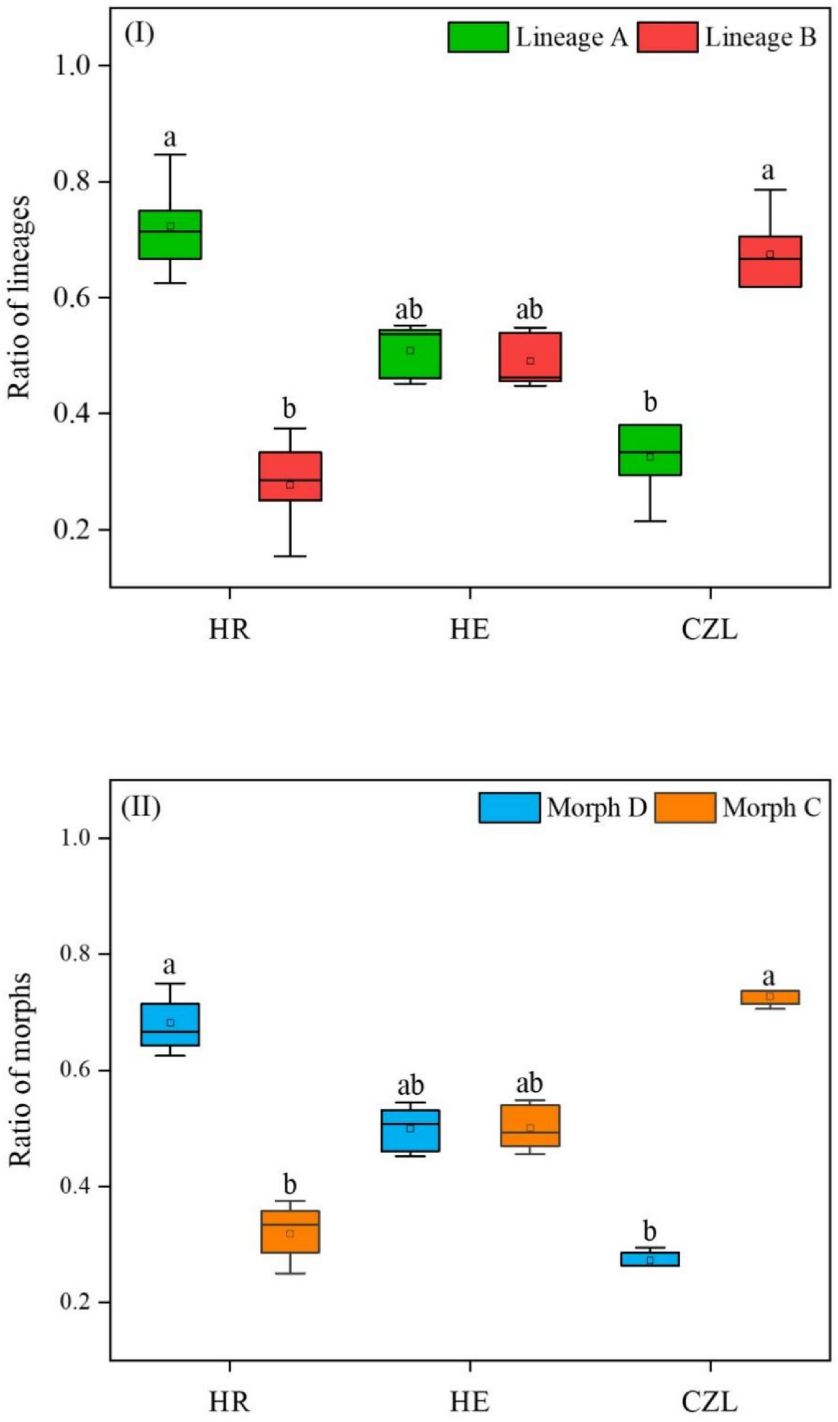
Density ratios of Lineages A and B (I), and Morphs C and D (II) of *Corbicula fluminea* in the Huaihe River (HR), the Huaihe Estuary (HE), and the Chengzi Lake (CZL) of the Hongze Basin. Different lowercase letters indicate significant differences between lineages and between morphs within each habitat or same lineage/morph among habitats (*p* < .05). The color of the box represents lineages and morphotypes (Lineage A: green, Lineage B: red, Morph C: orange, and Morph D: blue).

The density ratio between Lineages A and B, and Morphs C and D varied among the habitats (Figure 5). Lineage A accounted for 72.32% of all collected specimens in HR and was significantly higher than that of Lineage B (Kruskal–Wallis test: *χ* ^2^ = 21.930, *df* = 1, *p* = .000); there was no significant difference in the ratio between Lineages A and B in HE (Kruskal–Wallis test: *χ* ^2^ = 0.105, *df* = 1, *p* = .746); furthermore, Lineage B accounted for 67.48% of all collected specimens in CZL and was significantly higher than Lineage A (Kruskal–Wallis test: *χ* ^2^ = 9.843, *df* = 3, *p* = .002). Patterns of the density ratio between Morphs D and C were in agreement with patterns of Lineages A and B among the sampling sites (Figure 5).

## 4 DISCUSSION

Our results revealed two divergent groups in the Hongze Basin in regard to patterns of morphological and genetic variation in *C. fluminea*. Lineages A and B overall corresponded to Morphs D and C, and were, respectively, dominant in either lotic or lentic habitats. Additionally, both genetic lineages had the highest overall densities in the ecotone habitat. These results indicate a close correlation between morphological and genetic divergence and habitats for *C. fluminea* in the Hongze Basin.

Two lineages with significant genetic divergence between them were identified, corresponding to the two distinct morphotypes identified in a previous study for the lake (Li et al., 2022). Six individuals with 15 ridges/cm_sh_ had intermediate shell sculpture and showed inconsistent results between the morphological and genetic classifications. Whether morphological and genetic classifications correspond to each other exactly remains controversial (Sousa et al., 2007; Von Rintelen & Glaubrecht, 2006). Most studies have suggested that this inconsistency mainly contributes to the plasticity of morphological characteristics for the species when coping with different habitat conditions (Marin et al., 2016; Paolucci et al., 2014; Sousa et al., 2007). In our study, the shell sculpture pattern with 15 ridges/cm_sh_ was the threshold used to assign individuals to two morphotypes according to Li et al. (2022). The individuals with intermediate shell sculpture, assigned to Morph C, were all distributed in the lentic habitat and had the same genetic grouping as Morph D. These results seem to support the inference of morphological plasticity (see Section 4), with more data needed for confirmation. The ecological speciation hypothesis predicts that divergent processes in both morphological and genetic properties are likely to vary continuously and correspond to different morphotypes or lineages (Ravinet et al., 2018; Twomey et al., 2016). Excluding the intermediate shell sculpture, our results established a distinct correlation between morphotypes and genetic lineages, and indicate that *C. fluminea* may have experienced a series of divergence phases in the region regarding morphological and genetic properties.

Populations can cope with different habitat environments through phenotypic plasticity, and isolated populations among different habitats have a low possibility of exchanging individuals or gene flow, allowing for genetic differentiation to occur (Berner et al., 2008; Reusch et al., 2001). However, genetic divergence of *C. fluminea* is likely not related to patterns of geographic isolation or isolation by environment in this study. Because individuals of the two genetic lineages were simultaneously sampled at the same locations in our study, it means the two groups occur without geographic separation and barriers to gene flow or environmental dissimilarity that could restrict gene flow of *C. fluminea* populations. Many factors can structure genetic and phenotypic divergence of natural populations, such as sexual and natural selection, genetic drift, and geographic and temporal isolation (Slatkin, 1987; Wang & Summers, 2010). We have no data to confirm crucial factors for the divergence of *C. fluminea*, which may need genome‐wide sequence data, and specific design of controlled experiments to further explore patterns.

Despite co‐occurrence in statistically equivalent numbers in ecotone habitats, our results also revealed that the two lineages of *C. fluminea* had clear spatial distribution patterns in the Hongze Basin. Lineages A and B were dominant in the lotic and lentic habitats, respectively. These patterns could indicate that Lineages A and B may not prefer lotic or lentic sites (because of lower density compared with ecotone sites), but each lineage may be more tolerant to lotic and lentic habitats, respectively. Except for the six individuals with intermediate shell sculpture, genetic lineages detected in our results distinctly corresponded to two morphotypes (Li et al., 2022). The two morphotypes have different shell sculpture patterns that likely have crucial effects on their sedentary lifestyle. Individuals of Morph C have more numbers of, closely spaced, and less elevated ridges that may facilitate rapid burrowing into the substrate and be disadvantageous for holding its position when facing fast and turbulent water velocity (Germann et al., 2014; Hornbach et al., 2010; Lezin & Flyachinskaya, 2015). Individuals of Morph D have fewer numbers of, evenly spaced, and highly elevated ridges that have an anchoring advantage in the lotic habitat, but pay higher cost in burrowing and feeding for their sedentary lifestyle. It has been demonstrated that aquatic gastropods (such as the freshwater snail *Potamopyrgus antipodarum*; Thorson et al., 2017) and mussels (such as the Asian freshwater bivalve *Limnoperna fortune*; Montalto & Rojas Molina, 2014) change the morphological characteristics of shell to cope with the trade‐off between the lotic and lentic habitats, which is similar to patterns of dominance of two *C. fluminea* morphotypes in the lotic and lentic habitats in this study. Furthermore, distribution patterns of different morphotypes of *C. fluminea* in the lotic–lentic system may be mainly manifested by tolerant capacities to stressors in the trade‐off, which can be indicated by the highest densities of both morphotypes in the ecotone habitat. Generally, our study indicated a correlation among morphotypes and genetic lineages with different habitats for *C. fluminea* in the Hongze Basin, although deterministic mechanisms for the correlation need further clarification.

The two revealed genetic lineages in this study may have important implications for the management of fishery resources. In fisheries management, *C. fluminea* has been managed as a single management unit for a long time in the Hongze Lake (Bi et al., 2014). The same management strategy has also been implemented for the bivalve in other waters (Gorman et al., 2011; Zeng et al., 2023). Significantly divergent populations are not conducive to sustainable utilization of resources under a single management strategy (Reiss et al., 2009). Cod (*Gadus morhua*) consists of several genetically divergent populations and had been managed as a single management unit in Newfoundland and Labrador waters (Sterner, 2007). This resulted in collapse of the cod fisheries and slow recovery from the early 1990s in the waters. Our study suggests two lineages should be considered as different management units, which means that all measures in fisheries, such as total allowable catch, minimum catch standards, release, and translocation, should respect and design individual‐based schemes for each lineage of *C. fluminea*.

Our results revealed that Lineage A has a close monophyletic and morphological relationship with the invasive populations and is more tolerant to the lotic habitat. The introduction and dispersal of larvae of *C. fluminea* are often facilitated by transport in water currents (Hoyer et al., 2015; Modesto et al., 2023). Lineage A had the highest density in the ecotone, indicating it does well in transitional conditions. One key challenge in invasive species management and prevention is the variability of freshwater environments and the need for monitoring of different local habitats (Stringer & Prendergast, 2023). In invasive management for this species, these results suggest that attention should be paid to the dispersal of pioneer individuals in the lotic and ecotone habitats when monitoring, prevention, and control of invasive *C. fluminea* (Paganelli et al., 2018; Sedlacek & Schoenebeck, 2012). In addition, *C. fluminea* in different habitats also have different morphotypes (Hünicken et al., 2022; Li et al., 2022), which may result in different reproductive characteristics. *Corbicula fluminea* displays behavior that promotes successful invasions, which can be explained by their reproduction/life‐history traits (Yokoyama, 2019). This species includes reproductive strategies for both sexual lineages of native Asian and asexual lineages of invasive American and European lineages (Pigneur et al., 2011, 2014). Lineage A had a close genetic relationship with invasive populations, however Lineage B had a close relationship with the historical origin of those populations. It will be necessary to compare their biological and ecological characteristics of these two lineages (Modesto et al., 2023), which may be helpful to reveal the invasive mechanisms and patterns of *C. fluminea*.

In summary, *C. fluminea* has morphologic and genetic divergence that is associated with different habitats in our study. Individuals of Lineages A and B each dominate lotic and lentic habitats, respectively, but have highest population densities in the ecotone. This opens doors for researchers to further explore what are the actual advantages of these different morphotypes in different habitats. Future research might also focus on the different lineages in terms of feeding, growth, and reproductive characteristics. The bivalve has been found to have a few morphologically and genetically inconsistent individuals in previous studies (Pfenninger et al., 2002; Pigneur et al., 2011). These individuals with intermediate characteristics should be further considered regarding speciation with hybrids (Bespalaya et al., 2018; Park et al., 2002). Future research could be followed up with genome‐wide data, three‐dimensional (3D) morphological scans, and specific design of controlled experiments to relate morphology to habitats.

## AUTHOR CONTRIBUTIONS

**Meixiang Jia:** Data curation (equal); formal analysis (equal); investigation (equal); methodology (equal). **Fei Cheng:** Conceptualization (equal); funding acquisition (equal); project administration (equal). **Jin Li:** Data curation (equal); formal analysis (equal). **Bjorn Victor Schmidt:** Supervision (equal); writing – review and editing (equal). **Yacheng Li:** Funding acquisition (equal); project administration (equal). **Songguang Xie:** Conceptualization (equal); funding acquisition (equal); methodology (equal).

## CONFLICT OF INTEREST STATEMENT

None declared.

## Supporting information


Appendix S1.–S3.

## Data Availability

Detailed information on the relevant samples and measured data are provided in Appendices S1–S3. Genetic data are uploaded to NCBI (National Center for Biotechnology Information), GenBank accession numbers: OR676346–OR676377.
