# Supplementary material for: Distribution patterns of the two genetic groups of Corbicula fluminea in a lotic–lentic system
Source: Ecol Evol. 2024 May 20;14(5):e11339. doi: 10.1002/ece3.11339 (PMC11106041; doi:10.1002/ece3.11339)
Supplement: Supplementary file 1 — Appendix S1.–S3. [file ECE3-14-e11339-s001.docx]

**Appendix S1** Shell pictures of Morphs C and D (the Hongze Basin); Forms A, B, C, and D (America) (Haponski and Foighil, 2019), Forms R, Rlc, and S (Europe) (Marescaux et al., 2010).


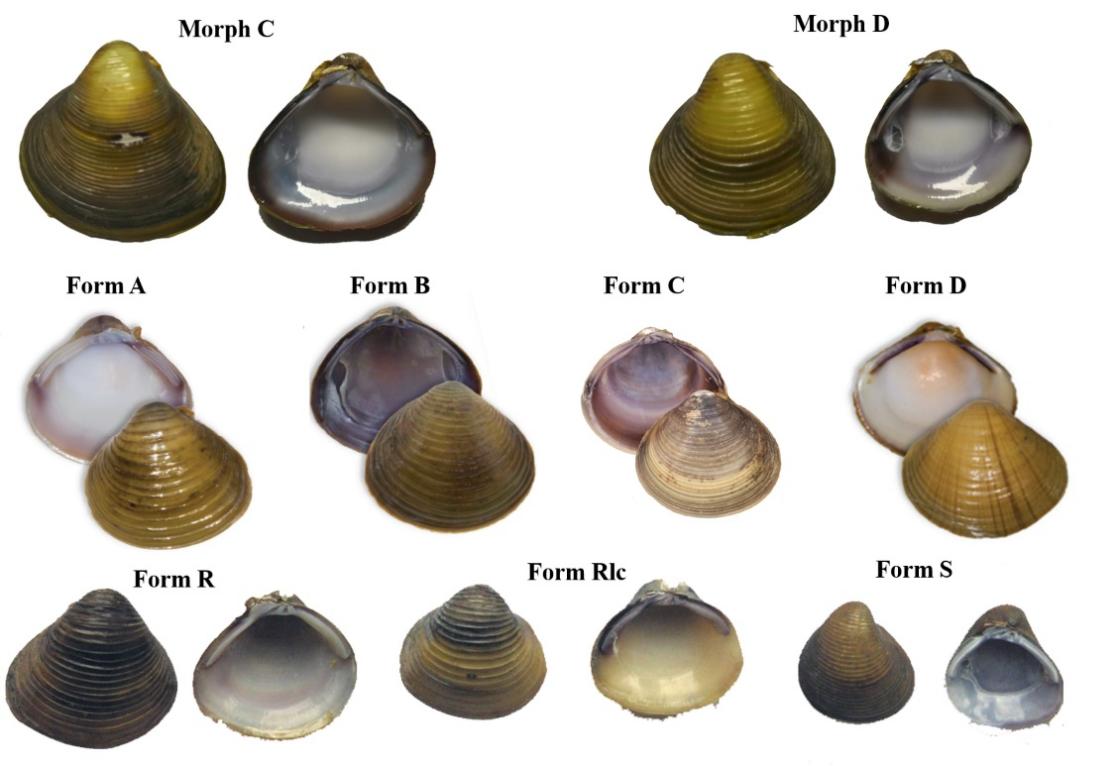


**Appendix S2** Habitat parameters (mean ± SD) of sampling sites in the Huaihe River (HR), the Huaihe Estuary (HE), and the Chengzi Lake (CZL) of the Hongze Basin

|  | HR | HE | CZL |
| --- | --- | --- | --- |
| Temperature (^o^C) | 31.85 ± 1.06 | 31.62 ± 0.88 | 31.04 ± 1.56 |
| Water depth (m) | 2.32 ± 0.65 | 2.00 ± 0.23 | 2.19 ± 0.27 |
| Transparency (cm) | 19.60 ± 5.34 | 13.80 ± 2.28 | 13.43 ± 5.59 |
| Conductivity (μs/cm) | 705.81 ± 159.81 | 583.00 ± 47.04 | 737.71 ± 72.23 |
| Dissolved oxygen (mg/l) | 10.09 ± 2.14 | 8.57 ± 2.84 | 8.02 ± 1.36 |
| pH | 9.59 ± 0.23 | 9.29 ± 0.28 | 8.65 ± 0.68 |
| Turbidity (NTU) | 116.95 ± 58.66 | 111.92 ± 59.78 | 137.71 ± 115.95 |
| Flow velocity (cm/s) | 0.41 ± 0.15 | 0.42 ± 0.18 | 0.15 ± 0.05 |
| Salinity (‰) | 0.26 ± 0.04 | 0.29 ± 0.06 | 0.34 ± 0.02 |

**Appendix S3** Polymorphic indices of Lineage A, Lineage B, Morph C, and Morph D of *Corbicula fluminea* in the Hongze Basin

| Group | Lineage A | Lineage B | Morph C | Morph D |
| --- | --- | --- | --- | --- |
| Specimen number | 53 | 36 | 42 | 47 |
| Haplotypes | 23 | 14 | 18 | 22 |
| Polymorphic sites | 35 | 19 | 77 | 34 |
| Haplotype diversity | 0.87 ± 0.04 | 0.87 ± 0.03 | 0.90 ± 0.03 | 0.87 ± 0.04 |
| Nucleotide diversity | 0.54 × 10^-2^ ± 0.14 × 10^-2^ | 0.42 × 10^-2^ ± 0.06 × 10^-2^ | 2.47 × 10^-2^ ± 0.63 × 10^-2^ | 0.48 × 10^-2^ ± 0.13 × 10^-2^ |
| Nucleotide differences | 3.71 | 2.91 | 17.09 | 3.33 |
